# Intraoperative and postoperative complications of gynecological laparoscopic interventions: incidence and risk factors

**DOI:** 10.1007/s00404-021-06192-7

**Published:** 2021-08-21

**Authors:** A. C. Kaya, M. P. Radosa, J. S. M. Zimmermann, L. Stotz, S. Findeklee, A. Hamza, P. Sklavounos, F. Z. Takacs, G. Wagenpfeil, C. G. Radosa, E. F. Solomayer, J. C. Radosa

**Affiliations:** 1 Department of Gynecology, Obstetrics and Reproductive Medicine, Saarland University Hospital, Homburg/Saar, Germany; 2 Department of Gynecology and Obstetrics, Klinikum Bremen-Nord, Bremen, Germany; 3 Department of Gynecology and Obstetrics, Kantonsspital Baden, Baden, Switzerland; 4 Institute of Medical Biometry, Epidemiology & Medical Informatics, Saarland University Hospital, Homburg/Saar, Germany; 5 Department of Diagnostic and Interventional Radiology, University Hospital Carl Gustav Carus, TU, Dresden, Germany

**Keywords:** Laparoscopy, Gynecology, Postoperative complication, Minimally invasive surgery, Hysterectomy

## Abstract

**Purpose:**

The aims of this study were to determine the incidence of intraoperative and postoperative complications of laparoscopic gynecological interventions and to identify risk factors for such complications.

**Methods:**

All patients who underwent laparoscopic interventions from September 2013 to September 2017 at the Department of Gynecology, Obstetrics and Reproductive Medicine, Saarland University Hospital were identified retrospectively using a prospectively compiled clinical database. Binary logistic regression analysis was used to identify independent risk factors for intra- and postoperative complications.

**Results:**

Data from 3351 patients were included in the final analysis. Overall, 188 (5.6%) intraoperative and 219 (6.5%) postoperative complications were detected. On multivariate analysis, age [odds ratio (OR), 1.03; 95% confidence interval (CI) 1.01–1.04], surgery duration (OR, 1.02; 95% CI 1.02–1.03), carbon dioxide use (OR, 0.99; 95% CI 0.99–1.00), and surgical indication (all *p* ≤ 0.01) were independent risk factors for intraoperative and duration of surgery (OR, 1.01; 95% CI 1.01–1.02; *p* ≤ 0.01), carbon dioxide use (OR, 0.99; 95% CI 0.99–1.00; *p* ≤ 0.01), hemoglobin drop (OR, 1.41; 95% CI 1.21–1.65; *p* ≤ 0.01), and ASA status (*p* = 0.04) for postoperative complications.

**Conclusion:**

In this large retrospective analysis with a generally low incidence of complications (5.6% intraoperative and 6.5% postoperative complications), a representative risk collective was identified: Patients aged > 38 years, surgery duration > 99 min, benign or malignant adnex findings were at higher risk for intraoperative and patients with surgery duration > 94 min, hemoglobin drop > 2 g/dl and ASA status III at higher risk for postoperative complications.

## Introduction

Minimally invasive techniques have replaced a plethora of open surgical interventions [1]. Numerous studies have shown that laparoscopy offers many advantages over open surgery, such as reduced intraoperative blood loss, postoperative pain levels, hospitalization and faster recovery times, including for obese patients, general surgeries, and the treatment of benign and malignant gynecological diseases [2–6]. Although minimally invasive techniques are the gold standards in many fields of surgery, they are intra-abdominal procedures, and complications of laparoscopic interventions are not less severe [7]. Reported overall complication rates vary between different authors, with ranges of overall complication rates between 4–41.21%, possibly due to differences in study design and cohort size [8, 17–20]. In addition, a major problem in research on laparoscopic complications is the lack of standardized definition and recording of complications. The aim of this observational study was to determine the rates of intraoperative and postoperative complications of laparoscopic gynecological interventions, and to identify risk factors for the occurrence of these complications.

## Materials and methods

### Study design and participants

All patients who underwent laparoscopy at the Department for Gynecology, Obstetrics and Reproductive Medicine, Saarland University Hospital between September 2013 and September 2017 were identified retrospectively using a prospectively compiled institutional clinical database. All patients who underwent laparoscopic gynecological surgeries were included in this study. Those who underwent laparoscopically assisted vaginal hysterectomies and those for whom intervention or patient data were incomplete were excluded.

A gynecological fellow collected the study data by systemic chart review. Data on patient characteristics [age (years), height (cm), weight (kg), body mass index (BMI; kg/m^2^)], surgical parameters such as surgery duration (min), carbon dioxide (CO_2_) use (l) (defined as amount of carbon dioxide in liter insufflated from the insertion of the Veress needle to the removal of the last trocar), hemoglobin (Hb) drop (g/dl) (defined as difference between the last preoperative and the first postoperative hemoglobin control in g/dl), surgical indications [symptomatic uterine myoma, benign or malignant adnexal finding, cervical cancer, endometrial carcinoma, endometriosis, urogynecological indication, cervical intraepithelial neoplasia (CIN)], histological findings (benign/malignant), laparoscopic intervention difficulty according to Barakat [low–medium (levels I and II), medium high–high (levels III and IV)], American Society of Anesthesiologists (ASA) physical status, adhesiolysis and duration of postoperative hospital stay (days) were extracted [9, 10].

Surgical complications were recorded as intra- and postoperative. Bleeding, blood transfusion, organ injury, conversion to laparotomy, resuscitation and skin emphysema were defined as relevant intraoperative complications. Postoperative complications (all deviations from the normal postoperative course) occurring during a 6-week period after surgery were classified using the five-grade Clavien–Dindo system and as minor (levels I and II) and major (levels III to V) [8, 11].

### Procedures

All laparoscopies were performed by experienced consultants. All patients received perioperative antibiotic prophylaxis with cefuroxime (single shot, 1.5 g i.v.; Fresenius Kabi, Bad Homburg, Germany) and, if necessary, metronidazole (single shot, 500 mg i.v; Fresenius Kabi, Bad Homburg, Germany) and a perioperative gastric tube and indwelling urinary catheter until mobilization on the first postoperative morning. For perioperative thromboembolism prophylaxis, low-molecular-weight heparin (enoxaparin sodium, 40 mg; Sanofi, Paris, France) was injected daily during patients’ entire hospital stays [12]. For hysterectomies of benign and malignant indication, such prophylaxis was administered for 1 and 4 weeks, respectively. Laparoscopy was performed in lithotomy position using the four-port technique; specifics of the techniques used for different interventions have been described previously [12–16]. After surgery, all patients were taken to postoperative ward for 4 to 6 hours of monitoring vital signs, then moved to general ward; blood count control was performed on the first postoperative day. Before discharge from hospital, final examination with speculum insertion and transvaginal and kidney ultrasound was performed in all cases. Patients were advised to present to the emergency ward if any deviation from the normal postoperative course occurred.

### Statistical analysis

All data were collected with Microsoft Excel (Excel 2014, Microsoft Corporation, Redmond, WA, USA). The statistical analysis was performed using IBM SPSS (Version 25, IBM SPSS Inc., Chicago, IL, USA). For quantitative data, the Kolmogorov–Smirnov test was used to assess the normality of distribution. As the data were not distributed normally, medians and ranges were calculated and the Mann–Whitney *U* test was used for comparison. For categorical variables, absolute and relative frequencies were determined, and the Pearson chi-squared test was used for comparison. Patient characteristics and surgical parameters were compared according to the presence or absence of intraoperative and postoperative complications. Multivariate binary logistic regression with stepwise forward and backward selection was used to identify factors predicting the occurrence of these complications. A receiver operating characteristic (ROC) curve was used to vary the discrimination thresholds for quantitative risk factors.

## Results

### Patient characteristics and surgical parameters

Of 3409 patients identified from the database, 58 were excluded due to lack of information; thus, data from 3351 patients were examined in the present analysis. The patient characteristics and surgical parameters are presented in Table 1. The median patient age was 42 (range 9–95) years and the median BMI was 25.1 (range 15.6–62.5) kg/m^2^. The median duration of surgery was 82 (range 2–492) min. A median of 156 (range 7.4–2060) l carbon dioxide was used during surgery and a median hemoglobin drop of 1.0 (range − 4.2–8.5) g/dl occurred. The median length of hospital stay was 3 (range 0–75) days. The most common indication for surgery was a benign adnexal finding [*n* = 900 (26.9%)]. Malignancy was diagnosed in 421 (12.6%) cases, and 2930 (87.4%) laparoscopies were performed due to benign findings. Most [*n* = 2484 (74.1%)] interventions were of technical difficulty level II and most patients were classified with a Grade II ASA status [*n* = 2080 (62.1%)]. Adhesiolysis was performed during 1950 (58.2%) laparoscopies (Table 1).

**Table 1:** Patient characteristics and surgical parameters

Patient characteristics and surgical parameters (*n* = 3351)	
	Median (minimum–maximum)
Age (years)	42 (9–95)
Weight (kg)	68 (33–172)
Body mass index (kg/m^2^)	25.1 (15.6–62.5)
Operative time (min)	82 (2–492)
Carbon dioxide use (l)	156 (7.4–2060)
Hemoglobin drop (g/dl)	1 (− 4.2–8.5)
Length of hospital stay (days)	3 (0–75)
	*n* (%)
Indication	
Benign adnexal finding	900 (26.9%)
Uterus myomatosus	823 (24.6%)
Endometriosis	537 (16.0%)
Others	494 (14.7%)
Urogynecological	
Indication	162 (4.8%)
Endometrial cancer	151 (4.5%)
Cervical cancer	131 (3.9%)
Malignant adnexal finding	115 (3.4%)
CIN	38 (1.1%)
Malignancy	
Yes	421 (12.6%)
No	2930 (87.4%)
Barakat-classification	
Grade II	2484 (74.1%)
Grade III	461 (13.8%)
Grade IV	345 (10.3%)
Grade I	61 (1.8%)
ASA–classification	
Grade II	2080 (62.1%)
Grade I	880 (26.3%)
Grade III	386 (11.5%)
Grade IV	5 (0.1%)
Grade V	0 (0.0%)
Grade VI	0 (0.0%)
Adhesiolysis	
Yes	1950 (58.2%)
No	1401 (41.8%)

*CIN* cervical intraepithelial neoplasia, *ASA-Classification* American Society of Anaesthesiologists Classification (*n* = 3351)

### Intra- and postoperative complications

Intraoperative complications were recorded in 188 (5.6%) cases. Conversion to laparotomy was the most common such complication [*n* = 92 (2.7%)], followed by organ injury [*n* = 75 (2.2%)]. Organ injuries were recorded for the intestinal tract [*n* = 35 (1.0%)], bladder [*n* = 29 (0.9%)], ureter [*n* = 3 (0.1%)], blood vessel [*n* = 1 (0.02%)], and “others” [e.g., uterus, diaphragm; *n* = 7 (0.2%)]. Other intraoperative complications were bleeding [*n* = 1 (0.02%)], the need for blood transfusion [*n* = 17 (0.5%)] and resuscitation [*n* = 2 (0.1%)], and skin emphysema [*n* = 1 (0.02%)]. Conversion to open surgery was performed due to complications in 62 (1.9%) cases and suspected or confirmed malignancy in 30 (0.9%) cases.

Postoperative complications were recorded in 219 (6.5%) cases; 92 (2.7%) were minor and 128 (3.8%) were major. According to clinical manifestation, revision of postoperative hemorrhage and hematomas was applied most commonly [*n* = 41 (1.2%)], followed by the antibiotic treatment of infections, hematomas, and abscesses [*n* = 28 (0.8%)] and transfusion for anemia [*n* = 26 (0.8%)]. Twenty-three (0.7%) patients presented at the hospital due to the post-discharge exacerbation of pain, 13 (0.4%) patients had cardiopulmonary complications, 7 (0.2%) patients had ileuses treated conservatively with prokinetic stimulation, and 5 (0.1%) patients had ileuses requiring surgery. Minimal vaginal cuff dehiscence that could be treated conservatively was documented in 3 (0.1%) cases; 12 (4%) cases of dehiscence requiring re-intervention occurred. Eighteen (0.5%) patients with intestinal problems (e.g., peritonitis, anastomosis insufficiency, fistula) and four (0.1%) patients with bladder injury, ureteral lesion, or vesicovaginal fistula required surgical treatment. Nerve lesions and paresthesia were registered in five (0.1%) cases, trocar site hernia or infection were recorded in two (0.1%) cases, lymphocele was recorded in one (0.02%) case, and compartment syndrome was recorded in two (0.1%) cases. Wound healing disorders were treated conservatively in two (0.1%) cases and operatively in four (0.1%) cases. Eight (0.2%) abscess revisions were recorded. Three (0.1%) patients had multiorgan dysfunction and 12 (0.4%) patients experienced other types of adverse event (Table 2).

**Table 2:** Intra- and postoperative complications

Intra- and postoperative complications (*n* = 3351)	
Intraoperative complications	Total (%)
Yes	188 (5.6%)
No	3163 (94.4%)
Type of complication	
Conversion to laparotomy	92 (2.7%)
*Due to complication*	*62 (1.9%)*
*Due to dignity*	*30 (0.9%)*
Organ injury	75 (2.2%)
* Intestine*	*35 (1.0%)*
* Bladder*	*29 (0.9%)*
* Ureter*	*3 (0.1%)*
* Vessel*	*1 (0.02%)*
* Others*	*7 (0.2%)*
Blood transfusion	17 (0.5%)
Resuscitation	2 (0.1%)
Skin emphysema	1 (0.02%)
Bleeding	1 (0.02%)
Postoperative complications	
Yes	219 (6.5%)
No	3132 (93.5%)
Type of complication	
CD IIIb	90 (2.7%)
CD II	65 (1.9%)
CD I	27 (0.8%)
CD IVa	24 (0.7%)
CD IIIa	11 (0.3%)
CD V	3 (0.1%)
CD IVb	0 (0.0%)
Clinical manifestation of postoperative complications	
Bleeding/hematoma-revision	41 (1.2%)
Infection/antibiotics/hematoma/abscess	28 (0.8%)
Anemia requiring transfusion	26 (0.8%)
Pain/adhesions	23 (0.7%)
Intestinal injury/peritonitis/anastomosis insufficiency/fistula-operative therapy	18 (0.5%)
Cardiopulmonal/thrombosis/embolism	13 (0.4%)
Vaginal cuff dehiscence-operative therapy	12 (0.4%)
Others	12 (0.4%)
Abscess-operative therapy	8 (0.2%)
Ileus-conservative therapy	7 (0.2%)
Ileus-operative therapy	5 (0.1%)
Nerve lesion/paresthesia	5 (0.1%)
Bladder injury/ureter injury/vesicovaginal fistula-operative therapy	4 (0.1%)
Wound healing disorder-operative therapy	4 (0.1%)
Vaginal cuff dehiscence-conservative therapy	3 (0.1%)
Multiorgan disfunction/death	3 (0.1%)
Trocar site hernia/infection	2 (0.1%)
Wound healing disorder-conservative therapy	2 (0.1%)
Compartment syndrome/ limb ischemia	2 (0.1%)
Lymphocele	1 (0.02%)

*CD* Clavien Dindo (*n* = 3351)

### Associations of surgical parameters with intraoperative complications

The intraoperative complication rate differed significantly between patients with benign and malignant diagnoses (112 (59.6% vs. 76 (40.4%, *p* ≤ 0.01). It further differed according to surgical indication [uterine myomas (*n* = 44; (23.4% vs. benign adnexal findings (*n* = 35; (18.6% vs. malignant adnexal findings (*n* = 31; (16.5% vs. cervical cancer (*n* = 17; (9.0% vs. endometrial cancer ((*n* = 23; 12.2% vs. endometriosis (*n* = 16; (8.5% vs. urogynecological conditions ((*n* = 6; 3.2% vs. CIN (*n* = 0, (0% vs. other conditions ((*n* = 16, 8.5%, *p* ≤ 0.01]. In addition, the occurrence of intraoperative complications differed according to the laparoscopy complexity level [(I: 0 (0%) vs. II: 87 (46.3%) vs. III: 32 (17%) vs. IV: 69 (36.7%), *p* ≤ 0.01], ASA status [I: 27 (14.4%) vs. II: 109 (58%) vs. III: 51 (27.1%) vs. IV: 1 (0.5%), *p* ≤ 0.01] and BMI [underweight: 8 (4.3%) vs. normalweight: 79 (42.0%) vs. overweight: 38 (20.2%) vs. obesity I: 32 (17%) vs. obesity II: 12 (6.4%) vs. obesity III: 19 (10.1%), *p* ≤ 0.01].

Relative to those without intraoperative complications, patients with such complications were significantly older [42 (range 9–95) vs. 50 (range 21–87) years, *p* ≤ 0.01], with longer surgeries [79 (range 2–415) vs. 180 (range 24–492) min, *p* ≤ 0.01], greater CO_2_ use [153 (range 7.4–2060) vs. 255 (range 7.7–1373) l, *p* = 0.02], larger hemoglobin drops [1.0 (range − 2.2–8.5) vs. 1.5 (range − 4.2–5.7) g/dl, *p* ≤ 0.01], higher BMIs [25 (range 15.6–62.5) vs. 26.1 (range 16.3–59.5) kg/m^2^, *p* ≤ 0.01] and longer hospital stays [3 (range 0–72) vs. 7 (range 0–75) days, *p* ≤ 0.01]. The intraoperative complication rate did not differ with regard to performance of adhesiolysis (Table 3).

**Table 3:** Surgical parameters in correlation with intraoperative complications

Surgical parameters in correlation with intraoperative complications (*n* = 3351)			
	Intraoperative complications		*p*-value
	Yes	No	
	Total (%)		
Indication			≤ 0.01
Uterus myomatosus	44 (23.4%)	779 (24.6%)	
Benign adnexal finding	35 (18.6%)	865 (27.3%)	
Malignant adnexal finding	31 (16.5%)	84 (2.7%)	
Endometrial cancer	23 (12.2%)	128 (4.0%)	
Cervical cancer	17 (9.0%)	114 (3.6%)	
Endometriosis	16 (8.5%)	521 (16.5%)	
Others	16 (8.5%)	478 (15.1%)	
Urogynaecological indication	6 (3.2%)	156 (4.9%)	
CIN	0 (0.0%)	38 (1.2%)	
Malignancy			≤ 0.01
Yes	76 (40.4%)	345 (10.9%)	
No	112 (59.6%)	2818 (89.1%)	
Barakat-classification			≤ 0.01
Grade II	87 (46.3%)	2397 (75.8%)	
Grade IV	69 (36.7%)	276 (8.7%)	
Grade III	32 (17.0%)	429 (13.6%)	
Grade I	0 (0.0%)	61 (1.9%)	
ASA-classification			≤ 0.01
Grade II	109 (58.0%)	1971 (62.3%)	
Grade III	51 (27.1%)	335 (10.6%)	
Grade I	27 (14.4%)	853 (27.0%)	
Grade IV	1 (0.5%)	4 (0.1%)	
Adhesiolysis			0.39
Yes	115 (61.2%)	1835 (58.0%)	
No	73 (38.8%)	1328 (42.0%)	
Body mass index			≤ 0.01
Normalweight	79 (42.0%)	1470 (46.5%)	
Overweight	38 (20.2%)	878 (27.8%)	
Obesity I	32 (17.0%)	414 (13.1%)	
Obesity III	19 (10.1%)	112 (3.5%)	
Obesity II	12 (6.4%)	186 (5.9%)	
Underweight	8 (4.3%)	103 (3.3%)	

	Median (minimum–maximum)		
	Yes	No	
Age (years)	50 (21–87)	42 (9–95)	≤ 0.01
Body mass index (kg/m^2^)	26.1 (16.3–59.5)	25 (15.6–62.5)	≤ 0.01
Operative time (min)	180 (24–492)	79 (2–415)	≤ 0.01
Carbon dioxide use (l)	255 (7.7–1373)	153 (7.4–2060)	0.02
Hemoglobin drop (g/dl)	1.5 (− 4.2–5.7)	1,0 (− 2.2–8.5)	≤ 0.01
Length of hospital stay (days)	7 (0–75)	3 (0–72)	≤ 0.01

*CIN* cervical intraepithelial neoplasia, *ASA Classification* American society of anaesthesiologists classification (*n* = 3351)

### Associations of surgical parameters with postoperative complications

The postoperative complication rate differed significantly between patients with benign and malignant diagnoses (161 (73.5%) vs. 58 (26.5%), *p* ≤ 0.01), and further according to surgical indication [uterine myoma (*n* = 60; 27.4%) vs. benign adnexal findings (*n* = 35; 16%) vs. malignant adnexal findings (*n* = 20; 9.1%) vs. cervical cancer (*n* = 10; 4.6%) vs. endometrial cancer (*n* = 23; 10.5%) vs. endometriosis (*n* = 24; 11%) vs. urogynecological indications (*n* = 15; 6.8%) vs. CIN (*n* = 2, 0.9%) vs. other indications (*n* = 30, 13.7%), *p* ≤ 0.01. In addition, the occurrence of postoperative complications differed according to the laparoscopy complexity level [I: 3 (1.4%) vs. II: 128 (58.4%) vs. III: 36 (16.4%) vs. IV: 52 (23.7%), *p* ≤ 0.01], ASA status [I: 43 (19.6%) vs. II: 127 (58%) vs. III: 48 (21.9%) vs. IV: 1 (0.5%), *p* ≤ 0.01], occurrence of intraoperative complications (62 (28.3%) vs. 157 (71.7%, *p* ≤ 0.01), and performance of adhesiolysis (143 (65.3%) vs. 76 (34.7%, *p* = 0.03).

Relative to those without postoperative complications, patients with such complications were significantly older [42 (range 9–95) vs. 47 (range 15–88) years], with longer surgeries (80 (range 2–492) vs. 132 (range 27–350) min], greater CO_2_ use [150 (range 7.4–2060) vs. 230 (range 7.7–1158) l], larger hemoglobin drops [1.0 (range − 2.4–5.8) vs. 1.3 (range − 4.2–8.5) g/dl], higher BMIs (25 (range 15.6–62.5) vs. 26.1 (range 16.3–57.5) kg/m^2^], and longer hospital stays [3 (range 0–41) vs. 5 (range 0–75) days; all *p* ≤ 0.01] (Table 4).

**Table 4:** Surgical parameters in correlation with postoperative complications

Surgical parameters in correlation with postoperative complications (*n* = 3351)			
	Postoperative complications		*p*-value
	Yes	No	
	Total (%)		
Indication			≤ 0.01
Uterus myomatosus	60 (27.4%)	763 (24.4%)	
Benign adnexal finding	35 (16.0%)	865 (27.6%)	
Others	30 (13.7%)	464 (14.8%)	
Endometriosis	24 (11.0%)	513 (16.4%)	
Endometrial cancer	23 (10.5%)	128 (4.1%)	
Malignant adnexal finding	20 (9.1%)	95 (3.0%)	
Urogynecological indication	15 (6.8%)	147 (4.7%)	
Cervical cancer	10 (4.6%)	121 (3.9%)	
CIN	2 (0.9%)	36 (1.1%)	
Malignancy			≤ 0.01
Yes	58 (26.5%)	363 (11.6%)	
No	161 (73.5%)	2769 (88.4%)	
Barakat-classification			≤ 0.01
Grade II	128 (58.4%)	2356 (75.2%)	
Grade IV	52 (23.7%)	293 (9.4%)	
Grade III	36 (16.4%)	425 (13.6%)	
Grade I	3 (1.4%)	58 (1.9%)	
ASA-classification			≤ 0.01
Grade II	127 (58.0%)	1953 (62.4%)	
Grade III	48 (21.9%)	338 (10.8%)	
Grade I	43 (19.6%)	837 (26.7%)	
Grade IV	1 (0.5%)	4 (0.1%)	
Adhesiolysis			0.03
Yes	143 (65.3%)	1807 (57.7%)	
No	76 (34.7%)	1325 (42.3%)	
Body mass index			0.06
Normalweight	90 (41.1%)	1459 (46.6%)	
Overweight	61 (27.9%)	855 (27.3%)	
Obesity I	32 (14.6%)	414 (13.2%)	
Obesity III	17 (7.8%)	114 (3.6%)	
Obesity II	13 (5.9%)	185 (5.9%)	
Underweight	6 (2.7%)	105 (3.4%)	
Intraoperative complications			≤ 0.01
Yes	62 (28.3%)	126 (4.0%)	
No	157 (71.7%)	3006 (96.0%)	
Type of complication			
Laparoconversion	40 (18.3%)	52 (1.7%)	
Organ injury	13 (5.9%)	62 (2.0%)	
Blood transfusion	7 (3.2%)	10 (0.3%)	
Resuscitation	1 (0.5%)	1 (0.0%)	
Skin emphysema	1 (0.5%)	0 (0.0%)	
Bleeding	0 (0.0%)	1 (0.0%)	

	Median (minimum–maximum)		
	Yes	No	
Age (years)	47 (15–88)	42 (9–95)	≤ 0.01
Body mass index (kg/m^2^)	26.1 (16.3–57.5)	25.0 (15.6–62.5)	≤ 0.01
Operative time (min)	132 (27–350)	80 (2–492)	≤ 0.01
Carbon dioxide use (l)	230 (7.7–1158)	150 (7.4–2060)	≤ 0.01
Hemoglobin drop (g/dl)	1.3 (− 4.2–8.5)	1.0 (− 2.4–5.8)	≤ 0.01
Length of hospital stay (days)	5 (0–75)	3 (0–41)	≤ 0.01

*CIN* cervical intraepithelial neoplasia, *ASA Classification* American Society of Anaesthesiologists Classification (*n* = 3351)

### Risk factor analysis: multivariate analysis

On multivariate analysis, independent risk factors for the occurrence of intraoperative complications were age [odds ratio (OR), 1.03; 95% confidence interval (CI), 1.01–1.04], surgery duration (OR, 1.02; 95% CI, 1.02–1.03), carbon dioxide use (OR, 0.99; 95% CI, 0.99–1.00), and surgical indication (all *p* ≤ 0.01). In relation to the reference group (endometriosis), the intraoperative complication risk was significantly greater among patients with benign adnexal findings (OR, 2.58; 95% CI 1.02–6.49; *p* = 0.04) and malignant adnexal findings (OR, 4.68; 95% CI 1.58–13.56; *p* ≤ 0.01; Table 5). Independent risk factors for postoperative complications were the duration of surgery (OR, 1.01; 95% CI 1.01–1.02; *p* ≤ 0.01), carbon dioxide use (OR, 0.99; 95% CI 0.99–1.00; *p* ≤ 0.01), hemoglobin drop (OR, 1.41; 95% CI 1.21–1.65; *p* ≤ 0.01), and ASA status (*p* = 0.04; Table 6).

**Table 5:** Multivariate analysis of factors associated with the occurrence of intraoperative complications

Multivariate analysis of factors associated with the occurrence of intraoperative complications (*n* = 3351)			
	Odds Ratio	95% CI	*p*-value
Age (years)	1.03	1.01–1.04	≤ 0.01
Operative time (min)	1.02	1.02–1.03	≤ 0.01
Carbon dioxide use (l)	0.99	0.99–1.00	≤ 0.01
Indication (vs. endometriosis)			≤ 0.01
Malignant adnexal finding	4.68	1.58–13.56	≤ 0.01
Benign adnexal finding	2.58	1.02–6.49	0.04
Uterus myomatosus	2.11	0.94–4.74	0.07
Cervical cancer	0.83	0.29–2.31	0.72
Endometrial cancer	0.94	0.32–2.69	0.90
Urogynaecological indication	0.41	0.13–1.35	0.14
CIN	0.00	0.00–1.00	0.99
Others	1.88	0.69–5.09	0.21

*CIN* cervical intraepithelial neoplasia, *95% CI* 95% confidence interval, *vs.* versus (*n* = 3351)

**Table 6:** Multivariate analysis of factors associated with the occurrence of postoperative complications

Multivariate analysis of factors associated with the occurrence of postoperative complications (*n* = 3351)			
	Odds ratio	95% CI	*p*-value
Operative time (min)	1.01	1.01–1.02	≤ 0.01
Carbon dioxide use (l)	0.99	0.99–1.00	≤ 0.01
Hemoglobin drop (g/dl)	1.41	1.21–1.65	≤ 0.01
ASA-classification			0.04
I vs. II	1.05	0.70–1.58	0.81
I vs. III	1.76	1.05–2.95	0.03
I vs. IV	6.57	0.65–66.63	0.11

*ASA-Classification* American Society of Anaesthesiologists Classification, *95% CI* 95% confidence interval; vs. = versus (*n* = 3351)

### ROC analysis

ROC analysis revealed that surgery duration > 99.5 min (AUC 0.8) and age > 38 years (AUC 0.7) were appropriate thresholds for the intraoperative complication risk. For postoperative complications, the thresholds were surgery duration > 94.5 min (AUC 0.7) and hemoglobin drop > 2.05 g/dl (AUC 0.6).

## Discussion

In this large retrospective study based on prospective clinical data, the intraoperative and postoperative complication rates of laparoscopic gynecological surgeries were 5.6% and 6.5%, respectively. Patient age, duration of surgery, carbon dioxide use, and surgical indication were independent risk factors for intraoperative complications. These complications were more frequent in patients with benign and malignant adnexal findings than in those with endometriosis (reference group). Risk factors for postoperative complications were duration of surgery, carbon dioxide use, hemoglobin drop, and ASA status, with ASA grade III associated with a greater risk than ASA grade I. These low complication rates are similar to those obtained in other studies and confirm the safety of laparoscopic interventions. The standardization of surgical procedures and identification of risk collectives might help to further reduce complication rates.

A few previous major studies also focused on the incidence of complications in laparoscopic gynecological surgeries. Chapron et al. evaluated surgical complication rates of diagnostic and operative procedures (29,966 cases) and found a total rate of 0.46% [17]. Similarly, Härkki-Siren et al. evaluated the nationwide incidence of laparoscopic complications in a cohort of 70,607 patients and assessed a rate of 0.36% [18]. A higher incidence of complications was recorded in the analysis of Mac Cordick et al. with a rate of 2.9% out of 743 procedures and Leonard et al. with 3% in a cohort of 1033 patients [19, 20].

Two main explanations can be offered for the difference between these rates and those obtained in the present study.

First, in contrast to the previous studies, the Clavien–Dindo classification, which includes minor complications (e.g., need for analgesic or antibiotic treatment) and thus results in higher overall complication rates, was used in this study [11].

For instance, the postoperative complications considered by Chapron et al. would be classified as of Clavien–Dindo grades ≥ III; the exclusion of minor complications can be argued to have led to the underestimation of complication occurrence [17]. In a study from Radosa et al., in which the Clavien–Dindo classification was used, the total complication rate among 7438 laparoscopies was 13% [8].

Another possible explanation for the “increase” in complication rates is the development of laparoscopic techniques over time and their expanded application to more advanced procedures, with concurrent evolution of systems used to classify laparoscopic surgeries. The classification of Chapron et al. was used in most previous studies to characterize the complexity of laparoscopic interventions (as diagnostic, minor, major, and advanced) [17].

The “advanced” category of this classification encompasses levels III and IV of the Barakat classification used in this study. Advanced procedures comprised 9.4–11.5% of surgeries in the previous studies, and comparable (Barakat levels III and IV) procedures comprised 24.1% of surgeries in the present study.

As in this study, Mirhashemi et al. found that the surgical indication was an independent risk factor for intraoperative complications; they reported lower rates associated with endometriosis and ovarian cystectomy [21]. Similarly, Saidi et al. reported that ovarian cystectomy was associated with the lowest rate of common complications [7]. We found that endometriosis was associated with a lower rate of intraoperative complications than were benign and especially malignant adnexal findings. Significant correlations between malignancy and complications have been reported previously [9, 22]. The differences between these findings and ours may be explained by differences in study design; in the present study, we examined a large sample of patients undergoing a wide range of gynecological interventions.

Increasing age was an independent risk factor for the occurrence of intraoperative complications in this study. Three recent studies with designs similar to that of the present study yielded similar results. Age was an independent risk factor for complications of gynecological laparoscopic interventions in a sample of 1451 cases (along with previous radiation therapy and malignant diagnosis), and for bowel injury during hysterectomy in a cohort of 15,557 patients (along with endometriosis, and abdominal surgical approach) [9, 23]. Aside from surgery type, increasing age was the most important predictor of complications of laparoscopic gynecological surgery in a sample of 843 patients; the risk of operative injury or bleeding was five times greater among women aged ≥ 35 years than among those aged < 35 years [21]. In the present study, we found that intraoperative complications were more likely to occur in women aged > 38 years. Older age may be a significant risk factor in this context due to the overall higher incidence of comorbidities with increasing age.

Of note, malignancy was not identified as a risk factor in our analysis, as it was in a previous study [9]. Chi et al.’s sample included more surgeries with malignant (*n* = 724) than with benign (*n* = 691) indications, whereas the majority of interventions examined in the present study were performed due to benign indications [9]. Thus, this discrepancy may be attributable to the larger percentage of malignant cases in Chi et al.’s smaller cohort [9].

The duration of surgery (i.e., longer operative time) was an independent risk factor for intraoperative and postoperative complications in this study. Two previous studies yielded similar results. In one study, the operative time (along with arterial hypertension, chronic obstructive pulmonary disease, and ASA status) was an independent risk factor for postoperative complications of 9145 laparoscopic procedures performed for the treatment of endometrial cancer [24]. In the other study, the operative time was associated independently with 30-day complications after laparoscopic and robotic hysterectomies [28]. Longer operative times can be associated with greater intraoperative blood loss and thus greater intraoperative and postoperative transfusion requirements and an increased risk of surgical site infection [25, 26]. They can also result in increased postoperative pain and increased risks of deep vein thrombosis and pulmonary artery embolism [27].

The ASA status describes patients’ preoperative health and is a predictor of postoperative mortality and outcomes of laparoscopic and open surgeries [29]. In this study, ASA grade III was associated with a significantly greater risk of postoperative complications than was ASA grade I. Two previous studies yielded similar results. The ASA status was an independent risk factor for postoperative complications of laparoscopic procedures in a sample of 9145 patients with endometrial cancer, and higher ASA status was a significant predictor of postoperative complications and length of hospitalization [24, 30]. Dean stated that this factor was useful for preoperative risk assessment [30].

The postoperative complication risk was also associated with the hemoglobin drop in this study. This variable has been reported as an independent risk factor for postoperative complications of other types of procedure (e.g., [31]). The hemoglobin drop presumably correlates directly with intraoperative blood loss and thus a greater postoperative transfusion requirement (a Clavien–Dindo grade II complication).

Carbon dioxide use was a risk factor for intraoperative and postoperative complications in this study. ORs for this variable were < 1, indicating that risk increased with reduced carbon dioxide use. For intraoperative complications, this association may reflect the conversion of laparoscopy to laparotomy (with no carbon dioxide use). We could not find a report on a comparable assessment of the association of carbon dioxide use with complications in the literature.

Based on the findings of this study, we can identify a representative risk collective, who may benefit from close postoperative monitoring and surveillance. The implementation of a standardized approach to surgical management and procedures that reduces the surgery duration and involves preoperative hemoglobin optimization might contribute further to the prevention of complications. Patients with ASA statuses > III might benefit from a multi-disciplinary approach to the preoperative optimization of health status.

### Limitations

This study was conducted at a single center, which limits the generalizability of the findings. However, the inclusion of a large number of patients in the analysis helps to minimize this limitation. In addition, possible bias in the recording of surgical complications was avoided by having an advanced medical student (rather than a layperson) perform data acquisition using a standardized classification system [32]. In addition, although many studies have revealed a significant correlation between surgeon expertise and the occurrence of complications [18, 19], we did not consider this variable as a possible risk factor in our analysis. However, highly qualified and experienced surgeons perform all laparoscopic procedures at our institution.

## Conclusion

In this large, single-center study, the overall complication rate for laparoscopic gynecological surgeries was low and independent risk factors for such complications were identified. Patients aged > 38 years, surgery duration > 99 min, benign or malignant adnex findings were at higher risk for intraoperative and patients with surgery duration > 94 min, hemoglobin drop > 2 g/dl, ASA status III at higher risk for postoperative complications. The findings can be used to develop strategies aiming to further reduce intraoperative and postoperative complications of these procedures.

## Data Availability

The dataset used and analyzed during the current study is available from the corresponding author on reasonable request.
